# The Effect of Terpenoid Natural Chinese Medicine Molecular Compound on Lung Cancer Treatment

**DOI:** 10.1155/2021/3730963

**Published:** 2021-12-16

**Authors:** Heng Sun, Lijia Zhang, Bowen Sui, Yu Li, Jun Yan, Peng Wang, Ye Wang, Songjiang Liu

**Affiliations:** ^1^The First Affiliated Hospital of Heilongjiang University of Chinese Medicine, Department of Oncology, Harbin, China; ^2^Heilongjiang University of Chinese Medicine, Harbin, China; ^3^The First Affiliated Hospital of Heilongjiang University of Chinese Medicine, Department of Oncology Ethics Office, Harbin, China; ^4^The First Affiliated Hospital of Heilongjiang University of Chinese Medicine, Department of Oncology Respiratory, Harbin, China; ^5^The First Affiliated Hospital of Heilongjiang University of Chinese Medicine, Academic Affairs Section, Harbin, China

## Abstract

Among all malignant tumors in the whole universe, the incidence and mortality of lung cancer disease rank first. Especially in the past few years, the occurrence of lung cancer in the urban population has continued to increase, which seriously threatens the lives and health of people. Among the many treatments for lung cancer, chemotherapy is the best one, but traditional chemotherapy has low specificity and drug resistance. To address the above issue, this study reviews the five biological pathways that common terpenoid compounds in medicinal plants interfere with the occurrence and development of lung cancer: cell proliferation, cell apoptosis, cell autophagy, cell invasion, metastasis, and immune mechanism regulation. In addition, the mechanism of the terpenoid natural traditional Chinese medicine monomer compound combined with Western medicine in the multipathway antilung cancer is summarized.

## 1. Introduction

The latest research has shown that lung cancer is the most common form of the malignant tumor whose morbidity and mortality have increased year by year in recent years. It has become the major cause of death due to cancer around the globe [1, 2]. According to the latest report, in 2018, approximately 18.1 million people worldwide were affected by different types of cancer. Among them, the number of deaths caused by lung cancer is about 2.1 million [3]. There has been advanced technology operated to treat lung cancer named as targeted therapy. The earliest targeted drugs used in clinics are mainly targeted at the epidermal growth factor and its receptor (EGF-EGFR) pathway, tyrosine kinase inhibitor, anti-VEGF monoclonal antibody, and VEGFR tyrosine kinase inhibitor (VEGFR-TKI) [4]. Since the beginning of 2000, multitarget drugs have rapidly developed [5]. Crizotinib is an ALK inhibitor, but it also targets LTK, CHEK2, FLT3, PHKG2, and RET. Nintedanib and sorafenib are multiple tyrosine kinase inhibitors targeting FGFR, PDGFR, and VEGFR [6]. Among them, gefitinib (Iressa) and erlotinib (trocet) are the most commonly used inhibitors. They are epidermal growth factor receptor inhibitors. Still, because of their selectivity for patients with gene mutations and the ease of medication, the occurrence of drug resistance has become the bottleneck of its widespread application [7, 8]. In the year 2020, there are a total of 89 small molecule targeted antitumor drugs that were approved by the American FDA and China's NMPA. These small molecule targeted at anticancer drugs are still at the challenging phase where they face drug resistance and low response rates [9].

Many active ingredients derived from herbal medicines of China have anticancer properties that include antiproliferative, proapoptotic, antimetastatic, and antiangiogenic effects [10]. These active ingredients can target the gene targets of the malignant proliferation of tumor cells, selectively induce tumor cell apoptosis without affecting normal cells, provide new research strategies for tumor treatment, and improve the treatment results of patients with NSCLC. New research emerges, including terpenoids, flavonoids, polyphenols, polysaccharides, and alkaloids. [11]. Among them, terpenoids are natural products derived from mevalonic acid (mevalonic acid, MVA). Terpenoids are composed of multiple isoprene (isoprene, C5) structural units, and the general formula is (C5H8) *n*, which is also the most common compound among natural products. It is present in a large quantity in the plant kingdom and has a large variety of styles. It has the potential to be a lead compound to develop efficient and new and safe antitumor drugs. At present, terpenoids with antitumor activity are classified into monoterpenes, sesquiterpenes, diterpenes, and triterpenes (Table 1).

In recent years, people have gradually realized that there are major effects of natural products on NSCLC, especially when it comes to improving anticancer activity and drug sensitivity. On the basis of this fact, the anticancer mechanism of natural small molecule compounds of terpenoid Chinese medicine on lung cancer has been summarized.

## 2. Cell Cycle Regulation and Inhibition of Tumor Cell Proliferation

An important feature of tumor cells is uncontrolled growth. The occurrence of tumors stems from changes in genetic material DNA (or genes), and this change is a multistep cellular process of multiple genetic changes. As mentioned above, cell cycle regulation is a delicate balance process, and any defects in this process may lead to changes in genetic information. The different phases of the cell cycle are highly accurately coordinated to ensure strict timing. For example, cyclins, CDK inhibitors, and other regulatory molecules need to be activated or degraded in time to ensure the regular operation of the cell cycle [12, 13]. In the course of normal cells into tumor cells, there may be cell cycle disorders, tumor cell invasion and metastasis, and drug insensitivity phenomenon [14]. Almost all tumors destroy the cell cycle regulation mechanism, leading to the characteristics of uncontrolled cell growth, blocked differentiation, and abnormal apoptosis [15]. In short, the destruction of the molecular regulation mechanism of the cell cycle, or the disorder of its upstream agent, will lead to an abnormal cell cycle. The following content is introduced, that is, the molecular mechanism of terpenoid natural TCM small molecules in treating lung cancer by inhibiting cell proliferation (Table 2).

Andrographolide (Andro) is the main active compound that is wholly spread in Andrographis paniculata, a kind of herb that is used as a natural remedy for various diseases [23, 24]. Studies have shown that Andro treatment can increase DNA fragmentation and reduce Na + -K + -ATPase activity, indicating *α*-subunit dysfunction and/or mitochondrial membrane damage, and also indicating mitochondrial dysfunction caused by AD, and reducing TGF-*β*1 and VEGF expression levels inhibits tumor cell proliferation and downregulates PCK to promote lung cancer cell apoptosis [16, 17].

The small molecule active substance of licorice is mainly glycyrrhizic acid with different chemical structure. Among them, there are many related types of research on glycyrrhizinic acid [25, 26]. Glycyrrhizic acid has various medicinal activities just like retinoic acid and steroids. Relevant studies have shown that 18*β*-GA can inhibit cell proliferation and induce NSCLC cell apoptosis at least in part [18, 19].

Glycyrrhetinic acid (GA) can inhibit cell lines of humans (A549 and NCI-H460), of which A549 is more sensitive than NCI-H460. Glycyrrhetinic acid activates p18/p16 to inactivate the CDK4/6-cyclin-D1/D3 complex. Glycyrrhetinic acid can activate p27/p21 to inactivate the CDK2-cyclin-E2 complex, thereby causing cell arrest in the G1 period. This condition can lead to the dephosphorylation of pRb in both cell types to regulate cell cycle progression and the inactivation of transcription factor 1 (E2F-1), a critical apoptotic transcription factor. GA is upregulated, and it has the ability to inhibit the proliferation of NSCLC cells via the emergency pathway of the endoplasmic reticulum. GA induces chaperones of ER, which reduces the synthesis of proteins [27].

Carnosic acid has various biological functions, which majorly include antibacterial, antioxidant, and antiproliferative activities [20]. Different treatment concentrations of CA can inhibit the proliferation/G one and G 2/M phases of IMR-90 and NCI-H460 cells [28].

There are two subclasses of ginsenosides, namely, protopanaxadiol (PPD) and protopanaxatriol (PPT) [21]. Related research has synthesized 13 kinds of ginseng diol derivatives through amino acids. Compared with PD, ginseng diol derivatives 3, 12, and 13 have a significant effect of inhibition on the cell proliferation of cancer cells. Among them, ginseng diol has the IC 50 value for A549 (IC 50 = 18.91 ± 1.03 microns) [19].

Astragaloside is the main active component of *Astragalus*, which is composed of many triterpene saponins, including astragaloside I-IV [29]. The study found that astragaloside IV if given in high dose can inhibit the growth of NSCLC cells (A549, HCC827, and NCI-H1299); while if it is present in low concentration, there will be no apparent cytotoxicity to the viability of the cell. In addition, astragaloside IV combined with medication majorly enhances the chemosensitivity of NSCLC cells [22].

## 3. Mechanisms of Inducing Apoptosis of Lung Cancer Cells

The process of apoptosis is a highly conservative suicide program of the cell. The body clears excess cells and dangerous cells through apoptosis to maintain homeostasis [30]. Inactivation of the apoptosis program can lead to the occurrence of autoimmune diseases and tumors. In cell cycle-monitoring point function inactivation, the obstruction of cell apoptosis can cause DNA damage to be carried into daughter cells, thereby increasing genome instability and causing cell malignancy [31]. Tumor cells can usually resist apoptosis [32]. At present, the primary strategies for targeted tumor therapy include the following: (1) the expression of the proapoptotic gene p53 is restored [33]; (2) the antiapoptotic gene Bcl-2 is inactivated [34]; (3) the sensitivity of tumor cell apoptosis is increased by regulating metabolism (Bax and Bak) [35]; and (4) biological therapy is related (antibody-directed therapy, immunotherapy, virus-based introduction of apoptosis-inducing factor p53, and proapoptotic miRNA) [36–38]. The following is introduced, that is, the molecular mechanism of terpenoid natural Chinese medicine small molecules to promote apoptosis of cancerous cells in the treatment of lung cancer (Table 3).

Atractylodes macrocephala I and III (ATL-I, III) are sesquiterpenoids derived from Atractylodes macrocephala. In in vitro studies, Bcl-2 and Bcl-XL in the expression of A549 cells treated with ATL-I decreased. In vivo studies have shown that ATL-I can effectively inhibit tumor growth in xenograft of mice by upregulating caspase-3 and caspase-9 [40].

Costunolide is known to be present in the germacranolide series. It was first extracted from the roots of Saussurea lappa Clarke [47]. Costus lactone is mediated by stress, and exposure activates the (UPR) signaling pathway. In addition, costus lactone induces ROS production, while the antioxidant N-acetylcysteine (NAC) is effective. It blocked ER stress and apoptosis activation, induced A549 cell apoptosis, and showed antitumor activity [41]. Costus lactone involves in the activation of caspase-3 and induces SK-MES-1 cell apoptosis [42].

Pachydermic acid (PA) can simultaneously induce apoptosis of NCI-H23 and NCI-H460 lung cancer. In addition, PA inhibits the NCI-H23 growth and xenograft cancers with an inability to cause toxicity in the host cell, inhibits proliferation of cells in tumor xenograft tissues, induces tumor cell apoptosis, and can also induce A549 cell apoptosis and destroy mitochondrial membrane potential [43, 44].

Polyphonic acid C (PPAC) is a triterpene compound derived from Poria cocos. PPAC induces cell apoptosis via the death receptor. In addition, the inhibition of the PI3-kinase/Akt signaling pathway and the enhancement of p53 activation indicate that this is an extra process to induce apoptosis [45].

Tubeimoside I (TBMS1), also known as tubeimoside A, is a triterpenoid isolated from the Bolbostemma paniculatum plant [48]. Tubeimoside I also has the ability to initiate apoptosis of lung cancer cells and destruct lysosome and mitochondrial pathways. One of its mechanisms is to induce DRP1-mediated mitochondrial breakage. It also disrupts lysosomal acidification by inhibiting V-ATPase, thereby blocking late-stage autophagic flux; this leads to the accumulation of reactive oxygen species (ROS). Cathepsin B upregulates Bax-mediated mitochondrial outer membrane permeability [46].

## 4. Autophagy and the Treatment of Lung Cancer

Cisplatin is an alkylating agent that has been approved for the treatment of ovarian, bladder, and lung cancers. However, accumulated evidence reveals the resistance to this platinum-containing drug, especially in lung cancer. The modulation of apoptosis-regulating proteins is wildly accepted as a major molecular mechanism associated with chemoresistance. Cisplatin-induced DNA damage generally results in the activation of p53 and, depending on the extent of damage and induces a variety of cellular responses including autophagy, apoptosis, and senescence. In this study, cisplatin induced autophagy to similar extents in H460 cells where p53 activity had been nullified by CRISPR/Cas9 (H460 crp53) as in the parental p53 wt H460 (H460wt) NSCLC cells and confirmed that cisplatin induced two different functional forms of autophagy, protective autophagy in the H460crp53 cells, and nonprotective autophagy in the H460wt cells. This autophagic switch was associated with greater sensitivity to cisplatin in the p53 wt cells. Of particular relevance, with pharmacologic or genetic inhibition of the cytoprotective autophagy in the p53 crp cells, the temporal decline in cell viability in response to cisplatin became virtually identical to that in the p53 wt cells through increased susceptibility to the promotion of apoptosis.

Andrographolide (Andro) is an active compound dispersed in Andrographis paniculata. Andrographis paniculata is a kind of herbal medicinal drug being utilized in Asia as a traditional medicine to treat many diseases [23, 24]. At present, cisplatin is used as first-line treatments for lung cancer. But, the effectiveness of this chemotherapy has the limitation that it is drug resistant. At the same time, the Andrographolide drug can inhibit cisplatin-induced autophagy and enhance cisplatin-mediated apoptosis [49]. In addition, Andro promotes the activation of Akt/mTOR signal by downregulating PTEN and inhibiting autophagy, resensitizing drug-resistant cells. Combination therapy with cisplatin and andrographolide can significantly prevent the growth of drug-resistant cells [50]. Therefore, Andro provides an ideal autophagy inhibitor candidate for clinical applications.

The inhibition of autophagy was found to improve the chemotherapy and its efficacy [51] GA-induced inhibition of cell proliferation and apoptosis are enhanced by inhibiting autophagy of the JNK pathway [52]. Ivy saponins are triterpenoids, and the inducible accumulation of ROS enhances cisplatin cytotoxicity by blocking autophagic flux. The proliferation of ROS promotes the proapoptotic effects of cisplatin and paclitaxel, and the ROS scavenger can eliminate the synergistic impact of N-acetyl-L-cysteine [53]. The above contents are described, that is, the molecular mechanism of terpenoid natural Chinese medicine small molecules to intervene in tumor cell autophagy in the treatment of lung cancer (Table 4).

## 5. Metastasis and Inhibition of Tumor Invasion

Metastasis linked with tumor invasion is a very complex phenomenon; multiple molecules affect tumor invasion-metastasis behavior from different aspects. Cell adhesion molecules are the key molecules to maintain the tissue structure, and they are also the main molecules that affect cell adhesion and motility [54]. The extracellular matrix is the tissue barrier for cancer cell invasion and metastasis. The enzymes responsible for degrading the extracellular matrix and their inhibitors are closely related to tumor metastasis [55]. Hepatocyte growth factor (HGF) and chemotactic factors are critical driving forces for tumor cell movement [56]. Promoting and inhibiting the balance between VEGF, PDGF, and TGF-*β* also directly affect the blood supply of tumors, which further affect metastasis [57–59]. An in-depth analysis of these various factors will help us more deeply understand the mechanism of tumor metastasis.

Curcumin is a kind of sesquiterpenoids with pharmacological activity, and it is an essential bioactive component mainly from plants of the genus Curcuma [60, 61]. Studies have been conducted to extract zedoary turmeric essential oil (EO-CZ) by steam distillation and to test the growth of cancerous metastasis in mice. The experimental results show that EO-CZ has effects of antiproliferation on B16BL6 and SMMC-7721 cells. MMP-7 is the smallest member of the matrix metalloproteinase. Extracellular matrix and substrate together constitute the first barrier in tumor metastasis, and its degradation is tumor invasion, and it is the critical link of transfer [62].

Tumor cells can induce tumor lymphatic endothelial cell (LEC) proliferation and lymphangiogenesis by expressing VEGF-C and VEGF-D to increase the number of lymphatic vessels and promote tumors. Lymphatic metastasis of cells can also increase the permeability of lymphatic vessels and the pressure of tumor interstitium, which facilitates the entry of tumor cells into blood vessels [63, 64]. At the same time, LEC can express chemokines and interact with surrounding microenvironmental mediators [65]. It can attract tumor cells expressing the corresponding receptor and promote tumor lymph node metastasis [66]. Some studies have extracted ten compounds, including costanolactone, by chromatography using bioassay-guided fractionation methods, which can inhibit TR-LE cells [67].

Oridonin is a natural compound that derives from ent-karate tetracyclic diterpene. It is first extracted from Isodon as a species [68]. It has the ability to inhibit the proliferation of H1688 cells and induce their apoptosis under high-dose treatment (20 *μ*mol/L). At the same time, oridonin (5 and 10 *μ*mol/L) inhibits the FAK-ERK1/2 signaling pathway without affecting cell proliferation and apoptosis [69].

Triptolide (TP) is a natural compound that has been isolated from the root of TP. Triptolide can change the expression of microRNA being utilized in cell movement and reduce the invasion of lung cancer cells. Triptolide reduces the expression of cell adhesion molecules, which leads to impaired downstream signal transduction and inhibits the formation of metastatic tumors in lung cancer mice injected with H358 cells [70]. In addition, TP combined with gefitinib can increase E-cadherin levels and inhibit cell proliferation. Therefore, a synergistic effect is produced to increase the resistance of A549 lung cancer cells to gefitinib and reverse the epithelial-mesenchymal transition (EMT) [71].

Astragaloside IV is the compound that has been extracted from *Astragalus* membranaceus [72]. AS-IV can inhibit the migration and invasion of A549, MMP-2, MMP-9, and integrin *β*1 and significantly reduce the level of E-cadherin. In addition, AS-IV can also considerably decrease the TGF-*β*1, TNF-*α*, and IL-6 levels [73] (Table 5).

## 6. Tumor-Immune Mechanism and Immunotherapy

The immune system is directly connected with the occurrence and formation of tumors in the body. The cancer antigens abnormally expressed by cells in the process of carcinogenesis are the core of tumor immunodiagnosis, and the body's various antitumor immune mechanisms are the theoretical basis for tumor immunotherapy. The body's antitumor mechanism is mainly based on cellular immune mechanisms. Different immune cells play an essential role in antitumor immunity. Antibodies' complements and cytokines are also effective molecules of tumor immunity [74]. Although the body has an antitumor immune mechanism, tumor cells can also evade immune attack through various mechanisms, eventually causing tumors to occur and develop in the body and even inhibit the body's immune function. Researchers, through the in-depth understanding of the body's antitumor immune mechanism, immune intervention on tumor patients, used various methods to reverse the body's immune status and activate the body's antitumor immune effect to achieve the purpose of treating tumors and prolonging survival [75].

**Figure 1:**
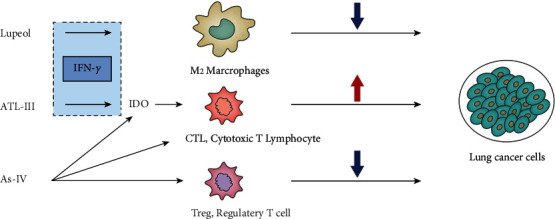
The influence of ATL-III, AS-IV, and lupeol on the immune mechanism of lung cancer.

Atractylenolide III (ATL-III) has been shown to inhibit the Jak3/Stat3 pathway-dependent IDO activation triggered by IFN-*γ*, and it is achieved by direct binding to the Jak3 protein [76]. IDO is also known to act as a checkpoint molecule that combines with cytotoxic T-lymphocyte antigen-4 and programmed cell death-1 to cause T-cell suppression after tumor transformation, in order to be prevented by the immune system from initiating the attack on cancer [77]. Experiments prove that Astragaloside IV (AS-IV) inhibits tumor growth in vivo experiments [78]. Studies have found that lupeol inhibits the growth of THP-1 by inhibiting the production of plasminogen activator inhibitor-1 (PAI-1) in H1299 cells. In addition, lupeol can inhibit the polarization of M2 macrophages and lead to the reduction in Lewis lung carcinoma (LLC) cell migration [79] (Figure 1).

## 7. Combination Medication and MultiChannel antitumor

**Figure 2:**
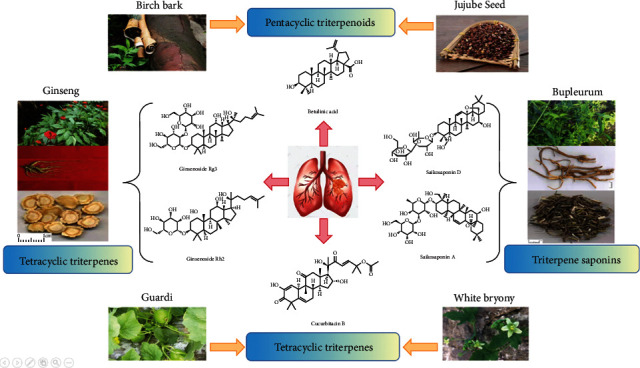
Herbal sources of terpenoid herbal compounds.

The use of multiple combinations as combined drugs to selectively target cancer cells has brought hope to a new generation of therapies (Figure 2).

### 7.1. Betulinic Acid

Betulinic acid (BA) can be considered ideal in treating lung cancer, and it provides a new treatment strategy for combination therapy. Studies have shown that the combination of 3 drugs (BA, ERKi, and HCQ) has a better therapeutic effect than a single treatment or therapy in xenotransplantation mouse models. Among them, betulinic acid, a natural compound, can inhibit cell proliferation and induce NSCLC cell apoptosis [80–84]. At the same time, the autophagy inhibition of hydroxychloroquine (HCQ) increased the response of lung cancer cells to the combination of BA and ERKi [80]. In addition, studies have proposed that betulinic acid (BA) and dichloroacetic acid (DCA) are chemically modified to synthesize a new combination drug Bet-CA, and its in vitro studies revealed that Bet-CA has apparent synergistic cytotoxicity to a broad spectrum of cancer cells, increased the production of reactive oxygen species (ROS), and promoted cells to undergo mitochondrial-mediated apoptosis; in vivo studies of Bet-CA showed tumor inhibition potential, and the clinically achievable dose will not produce any apparent toxicity [85].

Studies have suggested that BA treatment can induce apoptosis of paclitaxel-resistant human lung cancer H460 cells. In addition, BA can downregulate the expression of Bcl-2 and upregulate the expression of Bcl-2-related *X* (Bax) [86]. Studies have also suggested that sorafenib combined with betulinic acid strongly induces apoptosis of different NSCLC cells. In addition, this combination is not toxic to human peripheral blood lymphocytes. Compared with using two compounds alone, sorafenib combined with betulinic acid can induce apoptosis on different NSCLC cells (A549, H358, and A427) and eliminate the clonogenic activity [87, 88]. In vitro studies have shown that if, honokiol, ginsenoside Rh2, and betulinic acid are present in combination, they exhibit synergistic effects. Compared with the combination therapy group, the cisplatin group has obvious renal damage, and the combination therapy and the combination drug liposome therapy are safer [89].

### 7.2. Saikosaponin D

Saikosaponin D is a saponin extract extracted from Bupleurum (Umbelliferae), which can induce apoptosis to inhibit the proliferation of A549 [90]. Studies suggest that saikosaponin *D* inhibits the proliferation of lung cancer cells A549 (IC 50, 3.57 *µ*M) and H1299 (IC 50, 8.46 *µ*M) [91].

Saikosaponin A and saikosaponin D are two natural compounds derived from Bupleurum. At present, the combined application of natural small molecule compounds and chemotherapeutic drugs has become a research hotspot. Relevant studies have explored whether saikosaponins can sensitize the cytotoxicity of tumor cells induced by cisplatin. The results show that saikosaponin can make tumor cells [92].

In addition, studies have shown that saikosaponin D (SSD) and (EGFR-TKIs) gefitinib have an enhanced antitumor effect on NSCLC cells. It is related to the inhibition of the STAT3/Bcl-2 signaling pathway [93].

### 7.3. Cucurbitacin B

Cucurbitacin is a natural plant triterpenoid that belongs to the Cucurbitaceae. Cucurbitacin B (CuB) has antimetastatic, antiangiogenic, and antitumor immunity potential for NSCLC in vitro and in vivo [94, 95]. The expression of TPG and TSG can inhibit cell proliferation and induce apoptosis in NSCLC [96]. CUCs lead to the induction of programmed cell death, the inhibition of cell migration, and cell invasion. It can also regulate the expression of cyclin B1 to induce apoptosis and G2/M cell cycle arrest, thereby interfering with lung cancer metastasis [97, 98].

In the combined application of CuB and chemotherapeutics, the new semisynthetic derivatives of CuB (DACE) and three chemotherapeutic drugs, namely, cisplatin (CIS), irinotecan (IRI), and paclitaxel (PAC), can induce apoptosis in A549 cells. It regulates the cell cycle, has a synergistic antiproliferative effect, and does not reduce the proliferation of nontumor lung cells (MRC-5) [99]. In addition, the semisynthetic derivative of CuB, DACE (2-deoxy-2-amine-cucurbitacin E), and paclitaxel (PTX) showed potential in vitro synergistic antiproliferative effects in A549 cells [99]. In addition, CuB can reduce the proliferation of gefitinib-resistant (GR) PC9 cell lines by regulating the miR-17-5p/STAT3 axis [100].

### 7.4. Ginsenoside

Ginsenosides are one of the main components of ginseng and belong to typical terpenoids. Ginsenoside Rg3 reduces vascular endothelial growth factor expression and increases the ratio of CD4/CD8 T cells [101, 102]. We found that ginsenoside metabolite K (CK) can significantly enhance the expression and activity of p53 in the two lung cancer cell lines H460 and A549 induced by cisplatin and cooperate with cisplatin to inhibit tumor cell proliferation and induce apoptosis [103]. It decreased the antiapoptotic protein Bcl-2, which significantly increased the cells of NSCLC cell lines. Apoptosis substantially reduces the migration of NSCLC cells [104]. The combination therapy of ginsenoside Rg3 and gemcitabine can dramatically reduce the expression of VEGF and MVD, blood flow, and PSV signals in tumors, inhibit tumor growth, and prolong survival [105]. In addition, ginsenoside Rg18 can inhibit tumor cell proliferation by mediating *G* 1 phase block and intracellular ROS production in A549 human NSCLC cells, and p38, JNK, and NF-*κ*B/p65 [106, 107].

## 8. Discussion

In recent years, the application of small molecular compounds of natural Chinese medicine in antitumor research has gradually become a new focus of international cancer research. With the continuous in-depth analysis of the anticancer activity of natural Chinese medicines, many biologically active compounds derived from traditional Chinese medicines can be used to treat NSCLC. The role is gradually recognized. At present, plant-derived anticancer drugs account for about 32% of the total anticancer drugs, of which terpenoids account for a large proportion. Researchers use small molecular compounds of traditional Chinese medicine to influence the cell cycle, induce cell apoptosis, promote autophagy, inhibit tumor cell invasion and metastasis, improve tumor-immune microenvironment, and other ways to achieve tumor treatment and prolong patient survival. Even the combination of natural products and traditional drugs shows more vigorous anticancer activity and lower toxic and side effects than single chemotherapeutic drugs and targeted drug treatments. The application of Chinese herbal medicine with permanent history and economic benefits to cancer treatment, including lung cancer, may also bring new opportunities and challenges to cancer treatment. If traditional Chinese medicine can effectively reduce the cost of cancer treatment, improve the effect of cancer treatment, and apply it to the clinic, the cancer cure rate in China and even the world may be effectively controlled.

The multicomponent, multitarget, and multipathway characteristics of the active ingredients of traditional Chinese medicine also cast a veil of mystery on the study of the biological activity of conventional Chinese medicine monomers. In the future, we need to separate and extract effective small molecule compounds from traditional Chinese medicine by different scientific means so as to apply them to the treatment of tumors. There are still many challenges to overcome in clinical treatment. However, it is undeniable that in exploring new anticancer drugs, the combination of natural anticancer active ingredients of traditional Chinese medicine combined with chemotherapy and targeted therapy may become a unique choice for the treatment of patients with NSCLC.

## Figures and Tables

**Table 1:** Part of terpenoids and category of traditional Chinese medicine (TCM) for lung cancer treatment.

Category	For example
Terpenoid natural TCM molecular compound	Monoterpene, sesquiterpene, diterpene, and triterpene
A single TCM	Andrographis, licorice, carnosic, ginseng, astragalus, atractylodes, aucklandia, pachymic, rhizoma bolbostemmae, curcumol, tripterygium wilfordii, and bupleurum
TCM monomer	Andro, glycyrrhizic acid, glycyrrhetinic acid, carnosic acid, ginsenosides, astragaloside, atractylodes macrocephala, costunolide, pachymic acid, polyporenic acid, tubeimoside, ivy saponins, curcumol, oridonin, triptolide, lupeol, betulinic acid, saikosaponin, and cucurbitacin

**Table 2:** Molecular mechanism of terpenoid natural TCM small molecules in treating lung cancer by inhibiting cell proliferation.

Terpenoid natural TCM small molecules	Experimental model	Dose/concentration	Mechanism of action	Ref.
Andrographolide (AD)	H3255 NSCLC cells	AD (1.0, 2.5, or 5.0 *μ*M) for 24 h	Decreased in the na (+)-k (+)-ATPase activities; decreased VEGF and TGF-*β*1 level;	[16]
			inhibited protein kinase C activities in H3255 cells.	
			Released lactate dehydrogenase.	
			Increased DNA fragmentation level.	
Andrographolide (AD)	H3255 NSCLC cells	AD (1, 5, or 10 *μ*M) for 24, 48, or 72 h	Inhibited proliferation of H3255 cell; decrease in MMP-9 expression and activity.	[17]
18*β*-glycyrrhetinic acid (18*β*-GA)	A549, NCI-H460, and NCI-H23 NSCLC cells	18*β*-GA (80, 160, or 320 *μ*M) for 24 h	Decrease in cell proliferation induced by transfection with TxAS small-interfering RNA (siRNA);	[18]
			Inhibited TxAS and its initiated ERK/CREB signaling.	
Glycyrrhetinic acid (GA)	A549 and NCI-H460 NSCLC cells	GA (50, 25, 12.5, 6.25 or3.125 *μ*mol/l) 24, 48, or 72 h	Arrested cell cycle in G0/G1.;	[19]
			Inhibited (CKIs) (p18, p16, p27, and p21);	
			Inhibited cyclins (cyclin-D1, cyclin-D3, and cyclin-E);	
			Inhibited cyclin-dependent kinases (CDKs) (CDK4, CDK6, and CDK2).	
Carnosic acid (CA)	IMR-90 (human fetal lung fibroblasts) and NCI-H460 NSCLC cells	CA (40, 80, 160, 240, or 320 *μ*M) 24 h	Arrest at G0/G1 and G2/M phases.	[20]
13 panaxadiol (PD)	HepG-2 (human hepatoma cells), A549 NSCLC cells, MCF-7 (human breast cancer cells), or HCT-116 (human colon cancer cells)	PD (IC_50_ = 8.62 ± 0.23 *μ*M)	Inhibited cellular proliferation.	[21]
Astragaloside IV	A549, HCC827, NCI-H1299 NSCLC cells	Astragaloside IV high doses (10, 20, and 40 ng/ml) and low doses (1, 2.5, and 5 ng/ml)	Inhibited the mRNA and protein levels of B7-H3.	[22]

**Table 3:** The molecular mechanism of terpenoid natural Chinese medicine small molecules to promote tumor cell apoptosis in the treatment of lung cancer.

Terpenoid natural TCM small molecules	Experimental model	Dose/concentration	Mechanism of action	Ref.
Atractylodes macrocephala I (ATL-I)	A549 and HCC827 NSCLC cells	ATL-I (10, 20, and 40 *μ*M) for 48 h	Upregulation of caspase-3, caspase-9, and Bax;	[39]
			Downregulation of Bcl-2 and Bcl-XL	
Atractylodes macrocephala III (ATL-III)	A549 NSCLC cells	ATL-II I (1–100 *μ*M) for 24 h	Increased lactate dehydrogenase release;	[40]
			Modulated cell cycle on A549 cells;	
			Induced the release of cytochrome c;	
			Upregulation of Bax expression	
Costunolide	A549 NSCLC cells	Costunolide (0, 5, 10, 15, 25, or 30 *μ*M) for 24 h	Upregulation of GRP78 and IRE1*α* and the activation of ASK1 and JNK;	[41]
			Induced ROS generation;	
			Changed the antiapoptotic function of Bcl-2;	
Costunolide	SK-MES-1 human lung squamous carcinoma cells	Costunolide (40 and 80 *µ*M) for 24 h	Induced cell cycle arrest at G1/S phase;	[42]
			Upregulation in the expression of p53 and Bax;	
			Downregulation in the expression of Bcl-2 and activation of caspase-3;	
Pachymic acid (PA)	NCI-H23 and NCI-H460 lung cancer cells	PA (20, 40, or 80 *µ*M) for 24 h	Induced cell cycle arrest at G2/M phase;	[43]
			Induced ROS generation;	
			Activation of both c-Jun N-terminal kinase (JNK) and endoplasmic reticulum (ER) stress apoptotic pathways.	
Pachymic acid (PA)	A549 NSCLC cells	PA (0, 3, 10, 30, 60, 100, and 200 *µ*M) for 24 or 48 h	Inhibited anchorage-dependent and anchorage-independent A549 growth;	[44]
			Induced apoptosis of A549 cells;	
			Decreased IL-1 beta-induced activation of cPLA (2) and COX-2;	
			Suppressed IL-1 beta-induced activation of MAPKs;	
			Inhibited IL-1 beta-stimulated nuclear factor kappa B of NF-kB;	
Polyporenic acid C (PPAC)	A549 NSCLC cells	PPAC (0, 2, 6, 20, 60, or 200 *µ*M) for 24, 48, or 72 h	Suppressed PI3-kinase/Akt signal pathway;	[45]
			Enhanced p53 activation.	
Tubeimoside I (TBMS1)	NCI-H1299 and NCI-H1975 lung cancer cells	TBMS1 (0, 10, 20, and 30 *µ*M) for 24 h	Induction of DRP1-mediated mitochondrial fragmentation;	[46]
			Inhibited V-ATPase and blocked late-stage autophagic flux via;	
			Blocked the removal of dysfunctional mitochondria;	
			Induced ROS generation.	

**Table 4:** The molecular mechanism of terpenoid natural Chinese medicine small molecules to intervene in tumor cell autophagy in the treatment of lung cancer.

Terpenoid natural TCM small molecules	Experimental model	Dose/concentration	Mechanism of action	Ref.
Andrographolide (Andro)	A549 and Lewis lung cancer (LLC) cells	Andro (0, 7.5, 15, or *μ*M) for 0, 6, 12, or 24 h	Suppressed autophagy;	[49]
			Enhanced cisplatin-mediated apoptosis;	
Andrographolide (Andro)	A549 NSCLC cells	Andro (0, 7.5, 15, or *μ*M) for 0, 6, 12, or 24 h	Promoted the activation of the Akt/mTOR signaling by downregulating PTEN and suppressed autophagy;	[50]
			Resensitized the resistant cells to cisplatin-mediated apoptosis;	
Glycyrrhetinic acid (GA)	A549 and NCI-H1299 cells	GA (0, 40, and 60 *µ*M) for 24 h	Induced cytoprotective autophagy;	[52]
			Activated the IRE1*α*-JNK/c-Jun pathway;	
Hederagenin	NCI-H1299 and NCI-H1975 cells	Hederagenin (0, 25, 50, and 75 *µ*M) for 0, 2, 6, 12, or 24 h	Induced the increased autophagosomes;	[53]
			Upregulation of LC3-II and p62;	
			Indicated the impairment of autophagic flux	

**Table 5:** The molecular mechanism of terpenoid natural Chinese medicine small molecules to inhibit invasion and metastasis in the treatment of lung cancer.

Terpenoid natural TCM small molecules	Experimental model	Dose/concentration	Mechanism of action	Ref.
Essential oil of curcuma zedoaria (EO-CZ)	B16BL6 and SMMC-7721 cells;	EO-CZ (0, 5, 10, 20, 40, 80, and 120 *μ*M/ml) for 48 h	Inhibit B16BL6 and SMMC-7721 cell proliferation;	[62]
	HUVEC (human umbilical vein endothelial cells);		Inhibited CD34, MMP-2, and MMP-9;	
	Sprout vessels of Sprague-Dawley male rat aortic ring;			
Andrographis (Andro)	A549 NSCLC cells;	Andro (0, 1.0, 2.5, and 5.0 *µ*M) for 24 h	Inhibited the migration and invasion of A549 cells;	[63]
			Inhibited MMP-7 but not MMP-2 or MMP-9;	
			Suppressed on PI3K/Akt/AP-1 signaling pathway;	
Dehydrocostus lactone (DL)	Temperature-sensitive rat lymphatic endothelial (TR-LE) cells;	DL (0.01, 0.1, and 0.5 *µ*M) for 0, 6, 12, 24, or 48 h	Inhibition of the proliferation of TR-LE cells;	[67]
Oridonin	H1688 SCLC cells;	Oridonin (0, 2.5, 5, 10, 20, and 40 *µ*M) for 24 or 48 h	Inhibited cell migration;	[69]
	BEAS‐2B and HBE cells;		Not-affected cell proliferation and apoptosis;	
Triptolide (TP)	A549 NSCLC cells;	TP 10 nM for 48 h	Decreased migration and invasion of lung cancer cells;	[70]
	H460 and H358 cells;			
Triptolide (TP)	A549 NSCLC cells;	TP (1, 2, 4, 8, 16, and 32 ng/ml) f or 24, 36, or 48 h	Inhibited the migration and invasion of A549;	[71]
			Upregulated E-cadherin protein expression;	
			Downregulated the MMP9, Snail, and vimentin expression levels;	
Astagaloside IV (AS-IV)	A549 NSCLC cells.	AS-IV (0, 5, 10, and 20 *μ*M) for 24 h	Inhibited the migration and invasion of A549;	[73]
			Decreased the levels of MMP-2, MMP-9, integrin *β*1, TGF-*β*1, TNF-*α*, and IL-6 levels;	
			Related to the PKC-*α*-ERK1/2-NF-*κ*B pathway.	

## Data Availability

The data used to support this study are available from the corresponding author upon request.
